# In Vitro Antitumor Active Gold(I) Triphenylphosphane Complexes Containing 7-Azaindoles

**DOI:** 10.3390/ijms17122084

**Published:** 2016-12-11

**Authors:** Pavel Štarha, Zdeněk Trávníček, Bohuslav Drahoš, Zdeněk Dvořák

**Affiliations:** 1 Department of Inorganic Chemistry, Regional Centre of Advanced Technologies and Materials, Faculty of Science, Palacký University Olomouc, 17. listopadu 12, 771 46 Olomouc, Czech Republic; pavel.starha@upol.cz (P.S.); bohuslav.drahos@upol.cz (B.D.); 2 Department of Cell Biology and Genetics, Regional Centre of Advanced Technologies and Materials, Faculty of Science, Palacký University Olomouc, Šlechtitelů 27, 783 71 Olomouc, Czech Republic; zdenek.dvorak@upol.cz

**Keywords:** gold(I) complexes, 7-azaindole, triphenylphosphane, crystal structures, antitumor activity, in vitro

## Abstract

A series of gold(I) complexes of the general composition [Au(*n*aza)(PPh_3_)] (**1**–**8**) was prepared and thoroughly characterized (e.g., electrospray ionization (ESI) mass spectrometry and multinuclear nuclear magnetic resonance (NMR) spectroscopy). The *N*1-deprotonated anions of 7-azaindole or its derivatives (*n*aza) are coordinated to the metal centre through the *N*1 atom of their pyrrole ring, as proved by a single crystal X-ray analysis of the complexes [Au(*3I5Br*aza)(PPh_3_)] (**7**) and [Au(*2Me4Cl*aza)(PPh_3_)]·½H_2_O (**8**′). The in vitro cytotoxicity of the complexes **1**–**8** was studied against both the *cisplatin*-sensitive and -resistant variants of the A2780 human ovarian carcinoma cell line, as well as against the MRC-5 human normal fibroblast cell line. The complexes **4**, **5**, and **8**, containing deprotonated 3-iodo-7-azaindole, 5-bromo-7-azaindole, and 2-methyl-4-chloro-7-azaindole (*2Me4Cl*aza), respectively, showed significantly higher potency (IC_50_ = 2.8–3.5 µM) than *cisplatin* (IC_50_ = 20.3 µM) against the A2780 cells and markedly lower effect towards the MRC-5 non-cancerous cells (IC_50_ = 26.0–29.2 µM), as compared with the mentioned A2780 cancer cells. The results of the flow cytometric studies of the A2780 cell cycle perturbations revealed a G_2_-cell cycle phase arrest of the cells treated by the representative complexes **1** and **5**, which is indicative of a different mechanism of action from *cisplatin* (induced S-cell cycle phase arrest). The stability of the representative complex **8** in the water-containing solution as well as its ability to interact with the reduced glutathione, cysteine and bovine serum albumin was also studied using ^1^H and ^31^P-NMR spectroscopy (studied in the 50% DMF-*d_7_*/50% D_2_O mixture) and ESI+ mass spectrometry (studied in the 50% DMF/50% H_2_O mixture); DMF = dimethylformamide. The obtained results are indicative for the release of the *N*-donor azaindole-based ligand in the presence of the used biomolecules.

## 1. Introduction

Transition metal complexes offer a wide pallet of biological activities, including the anticancer or anti-inflammatory ones [1,2,3]. Several complexes, as it is known especially for platinum-based anticancer drugs [4], are in use in medical practice. Among the non-platinum complexes, several gold(I) complexes, such as triethylphosphane-(2,3,4,6-tetra-*O*-acetyl-*β*-d-thiopyranosato)gold(I) complex (*auranofin*; [Au(SAtg)(PEt_3_)]), are clinically used for the treatment of rheumatoid arthritis [5]. However, many gold(I) complexes (including *auranofin*) have recently been reported and frequently patented as antitumor active substances [5,6,7,8]. The published results revealed that various gold(I) complexes: (a) exceed activity of platinum-based drugs; (b) effectively overcome the tumour cell resistance to *cisplatin*; and (c) act through different mechanisms of action from the conventional platinum-based drugs. The above-mentioned *auranofin* successfully completed the Phase I and II of the clinical trials as the anticancer drug for the treatment of chronic lymphocytic leukaemia (CLL) [9,10], other clinical studies using *auranofin* for the treatment of different types of cancer (e.g., ovarian carcinoma) are in progress [11].

The mechanism of action of anticancer gold(I) complexes, although still not fully understood, seems to involve the inhibition of the glutathione reductase-like enzyme thioredoxin reductase (TrxR) [12]. However, it is well-known for *auranofin* and other substituted-phosphanegold(I) complexes, such as [AuCl(PPh_3_)], that these agents release their *S*-donor ligands (*auranofin* [Au(SAtg)(PEt_3_)]) or chlorido ([AuCl(PPh_3_)]) ligand under the physiological conditions to form [Au(PR_3_)]^+^ species which react easily with various biomolecules, such as albumin (AlbH) or glutathione (GSH) [5,13,14]. For example, the interaction of [AuCl(PPh_3_)] with the mixture of GSH and 5,5′-dithiobis-2-nitrobenzoic acid (dtnb) provided the [Au(PPh_3_)(SG)] adduct [15]. Interestingly, even the phosphane-based ligand (PR_3_) can be released when the original gold(I) complex interacts with the mentioned biomolecules, as proved for *auranofin* and [AuCl(PPh_3_)], to form species, such as [Au(SG)_2_]^−^ or [Au(Alb)_2_]^−^. On the other hand, several highly potent anticancer gold(I) complexes, e.g., [AuCl(NHC)] or [Au(NHC)(PPh_3_)]I were found to be inert to GSH [16,17,18], indicating that the above-mentioned ligand replacement is not most likely the necessary activation step of biologically active gold(I) complexes (NHC stands for *C*-donor 1,3-diethylbenzimidazol-2-ylidene carbene ligand).

**Figure 1:**
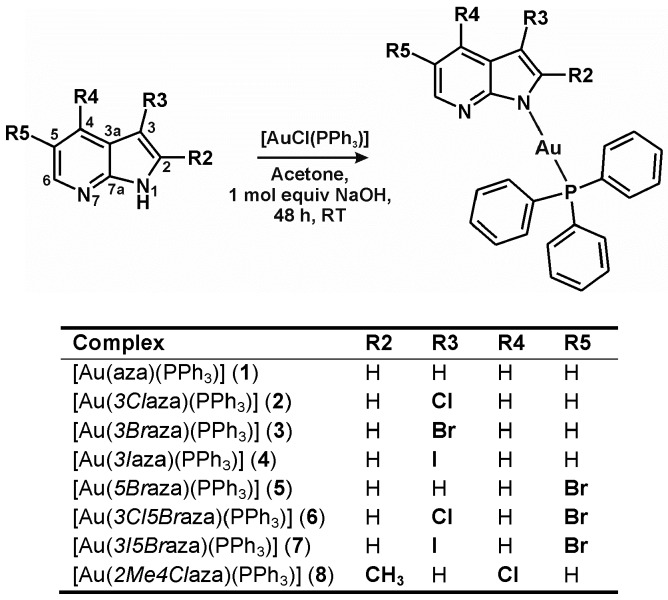
Synthetic pathway for the preparation of [Au(*n*aza)(PPh_3_)] (**1**–**8**), the structural formulas of the used 7-azaindoles (*n*Haza) and the studied complexes **1**–**8**, with atom numbering scheme and with a table summarizing the substituents of the 7-azaindole moiety for the used *n*Haza derivatives.

Recently, our research group reported the triphenylphosphanegold(I) complexes containing *N*6-benzyladenine-, hypoxanthine- and 9-deazahypoxanthine-derived *N*-donor ligands (HL), [Au(L)(PPh_3_)], which exhibited high cytotoxicity, ability to overcome acquired resistance to *cisplatin*, selectivity towards cancer cells over the normal ones and/or high anti-inflammatory activity [19,20,21,22]. In this work, we report on new complexes of the general composition [Au(*n*aza)(PPh_3_)] (**1**–**8**) containing the *N*1-deprotonated, C2-, C3-, C4-, and/or C5-substituted 7-azaindole-based ligands (*n*aza; Figure 1). 7-Azaindole itself (i.e., unsubstituted; Haza), as well as its halogeno-derivatives used in this work, are known as suitable ligands for various transition metal complexes [23,24], where they mostly act as electroneutral *N*7-coordinated ligand (e.g., [Pt(ox)(Haza)_2_], *cis*-[Pt(*3Cl*Haza)_2_Cl_2_] or [(*η^6^*-*p*-cym)Ru(Haza)Cl_2_]; ox = oxalate(2–), *p*-cym = *p*-cymene) [25,26,27] or *N*1-monodentate- (e.g., [Au(aza)(PPh_3_)]; **1** in this work), or *N*1,*N*7-bidentate-coordinated (e.g., [Cu_2_(μ-aza)_4_(dmf)_2_]) *N*1-deprotonated 7-azaindolate anions [28,29].

## 2. Results

### 2.1. Chemistry

In this work, we report on a series of eight triphenylphosphanegold(I) complexes of the general formula [Au(*n*aza)(PPh_3_)] (**1**–**8**), which were synthesized, thoroughly characterized in the solid state as well as in solution, and their in vitro cytotoxicity against both cancer and normal (i.e., non-cancerous) cell lines was studied, together with basic mechanistic aspects of their cytotoxic action. The complexes **1**–**8** were prepared in acetone from [AuCl(PPh_3_)] using a general procedure, as previously described in the literature for similar triphenylphosphanegold(I) complexes containing heterocyclic *N*-donor ligands different from 7-azaindole (Figure 1) [19,20,21,22,30]. The used 7-azaindoles (H*n*aza) were deprotonated in situ by an equimolar amount of NaOH. The products were isolated in good yields (≈70%) and acceptable purity (>95%), as proved by ^1^H-NMR and ^31^P-NMR spectroscopy; NMR = nuclear magnetic resonance. The studied substances are well-soluble in acetone, dimethylformamide (DMF), dimethyl sulfoxide (DMSO), chloroform, methanol, and ethanol, but the solubility in water was found to be very low. Complexes were stable in DMF-*d_7_* used for the NMR studies, because no changes were observed in the ^1^H-NMR and ^31^P-NMR spectra of **1**–**8** even after one week of standing at ambient temperature.

The *N*1–H hydrogen atoms of the used 7-azaindoles (*n*Haza), detected at ca. 12 ppm in their ^1^H-NMR spectra, were not found in the ^1^H-NMR spectra of the corresponding [Au(*n*aza)(PPh_3_)] complexes, as a consequence of deprotonation of these organic molecules by 1 M NaOH (Figures S1 and S2). The aromatic hydrogens of **1**–**8**, as well as the hydrogen atoms of the methyl group of the complex **8**, were found in the ^1^H-NMR spectra. The PPh_3_ hydrogen atoms were detected at ca. 7.7 ppm. All of the ^1^H-NMR aromatic C–H signals of the used 7-azaindoles shifted to higher fields after their coordination to the Au(I) atom, resulting in the negative coordination shift values (∆δ = δ_complex_–δ_ligand_; ppm). Regarding the ^13^C-NMR coordination shifts, high values (∆δ = 6.7–11.7 ppm), calculated for the C2 and C7a carbons adjacent to the N1 nitrogen atom (Figure 1), are consistent with the *N*1-coordination mode of the used *n*aza ligands. The *N*1-coordination mode was further proved by higher ^15^N-NMR coordination shift of N1 (48.0 ppm) than N7 (−0.8 ppm), as observed for the representative complex **5**. The ^31^P-NMR spectra of the studied complexes contained only one signal at ca. 33 ppm, assignable to the *P*-coordinated PPh_3_ molecule.

The positive mode electrospray ionization (ESI+) mass spectra of the complexes **1**–**8** contained, except for the most intensive peaks of the [Au(PPh_3_)_2_]^+^ species (100% relative intensity) at ca. 721.2 *m/z*, the characteristic molecular peaks of {[Au(*n*aza)(PPh_3_)] + H}^+^ (2%–10% relative intensity) and the peaks of [Au(PPh_3_)]^+^ and free {(H*n*aza) + H}^+^ (see Supplementary Materials and Figure S3). The peaks of the [Au(*n*aza)_2_]^−^ and (*n*aza)^−^ species were detected in the ESI− mass spectra of **1**–**8**.

Both the Fourier transform infrared (FTIR) and Raman spectra of the complexes **1**–**8** contained, similarly to the spectra of uncoordinated H*n*aza, the intensive peaks at 1574–1590 cm^−1^ and 1463–1481 cm^−1^, assignable to the ν(C–N)_ring_, and ν(C–C)_ring_ stretching vibrations, respectively [25,26]. Other peaks detected in the spectra at ca. 2900–3100 cm^−1^ belong to the ν(C–H)_ar_ of the *n*aza and PPh_3_ ligands [19,25,26,31,32]. The maxima of the peaks belonging to the ν(Au–N) and ν(Au–P) vibrations were centred at ca. 479–501 cm^−1^ and 319–330 cm^−1^, respectively [19,31,32].

### 2.2. Single Crystal X-ray Analysis

**Figure 2:**
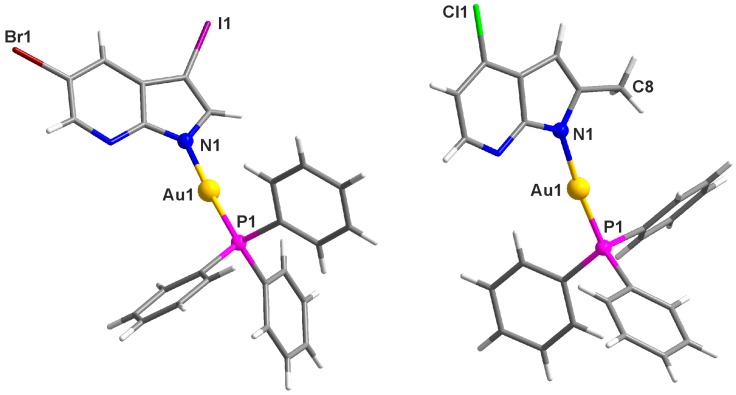
The molecular structures of [Au(*3I5Br*aza)(PPh_3_)] (**7**; **left**) and [Au(*2Me4Cl*aza)(PPh_3_)]·½H_2_O (**8**′; **right**), the ½H_2_O molecule of crystallization was omitted for clarity.

Two representative complexes, [Au(*3I5Br*aza)(PPh_3_)] (**7**) and [Au(*2Me4Cl*aza)(PPh_3_)]·½H_2_O (**8**′), were characterized by single crystal X-ray analysis. Crystal data and structure refinement parameters are given in Table 1. The molecular structures of **7** and **8**′ are depicted in Figure 2, while the selected bond lengths and angles can be found in Table 2.

Both the complexes **7** and **8**′ adopted the slightly distorted linear geometry. The Au1–N1 and Au1–P1 bond lengths, as well as the P1–Au1–N1 bond angle values (see Table 2) fall within the ranges of 1.97–2.12 Å (average of 2.06(3) Å) and 2.21–2.28 Å (average of 2.235(8) Å), and 168.4–179.6° (average of 175(2)°), respectively, in agreement with similar triphenylphosphanegold(I) complexes containing heterocyclic *N*-donor ligands, e.g., [21,22,28], deposited in the Cambridge Crystallographic Data Centre (CCDC; version 5.37 updated to May 2016 [33]). For the previously reported X-ray structure of [Au(aza)(PPh_3_)] (**1** in this work) [28], its P1–Au1–N1 bond angle value of 176.6° is consistent with the value of **7**, but differs markedly from that of **8**′. Regarding the bond lengths, Au1–N1 of **7** is higher than those of **8**′ (Table 2) and [Au(aza)(PPh_3_)] (**1** in this work; 2.033(4) Å) [28], while the Au1–P1 bond lengths of **7**, **8**′ (Table 2) and [Au(aza)(PPh_3_)] (2.2321(12) Å) do not differ markedly.

The crystal structure of **7** is stabilized by C–H···Br, C–H···I, C–H···C, C–H···N, C–H···π, and C···C non-covalent interactions (Table S1, Figure S4) [34,35]. In the case of **8**′, the O–H···N hydrogen bonds were detected between the N7 nitrogen atom of the 7-azaindole moiety and water molecule of crystallization, which together with C–H···O and C–H···C non-covalent contacts stabilize the crystal structure of this gold(I) complex (Table S1, Figure S5).

### 2.3. Aqueous Chemistry and Interaction Studies

#### 2.3.1. Solution Behaviour in a Water/DMF Mixture

**Figure 3:**
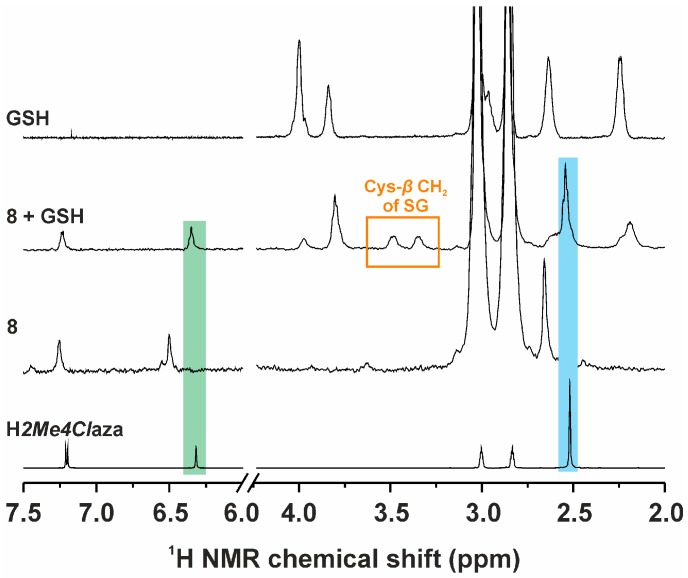
Proton nuclear magnetic resonance (^1^H-NMR) spectra of [Au(*2Me4Cl*aza)(PPh_3_)] (**8**) and its mixture with reduced glutathione (**8** + GSH) recorded after 48 h of standing at ambient temperature, given together (for comparative purposes) with the ^1^H-NMR spectra of free H*2Me4Cl*aza and GSH. All samples were studied in the 50% DMF-*d_7_*/50% D_2_O solution. Green: C3–H signal of H*2Me4Cl*aza; blue: CH_3_ signal of H*2Me4Cl*aza.

The representative complex **8** was found to be hydrolytically stable based on the ^1^H and ^31^P-NMR results, when dissolved in the 50% DMF-*d_7_*/50% D_2_O mixture. Only one set of ^1^H-NMR signals (e.g., C3–H signal at 6.50 ppm) and one ^31^P-NMR signal (34.8 ppm) were detected on the fresh solution, and no changes were observed even after 48 h of standing at ambient temperature. The positions of the ^1^H and ^31^P-NMR signals detected on the solution of **8** in the 50% DMF-*d_7_*/50% D_2_O mixture were different from those of free H*2Me4Cl*aza (C3–H signal at 6.32 ppm; Figure 3), PPh_3_ (^31^P-NMR signal at −4.7 ppm), the oxidized triphenylphosphane (O=PPh_3_; ^31^P-NMR signal at 32.4 ppm) or [AuCl(PPh_3_)] (^31^P-NMR signal at 34.2 ppm) dissolved in the same mixture of solvents (Figure S6).

#### 2.3.2. Interaction Studies with Biomolecules

The new ^1^H-NMR signals of Cys-*β* CH_2_ at 3.35 and 3.48 ppm, assignable to the *S*-deprotonated glutathione (i.e., SG) coordinated to the Au(I) atom through its sulphur atom, were detected immediately in the mixture of **8** and GSH (1 mol equiv), showing the formation of the [Au(PPh_3_)(SG)] adduct (Figure 3). The position of the ^31^P-NMR signal of **8** shifted from 34.8 ppm to 40.8 ppm, belonging most likely to the [Au(PPh_3_)(SG)] adduct (Figure S6). The characteristic C3–H signal of the free ligand (i.e., H*2Me4Cl*aza) was found at 6.35 ppm in the ^1^H-NMR spectra of the mixture of **8** and GSH (Figure 3). Neither new signals nor changes of intensity of the detected signals were observed after 48 h of standing at ambient temperature. Similar experiment performed with 5 mol equiv of GSH led to the same results (Figures S6 and S7). The peaks of the {[Au(PPh_3_)(SG)] + H}^+^ adduct (overlapped with {[Au(PPh_3_)(SG)] + 2H}^+^ in a 1.75:1.00 ratio) and the {[Au(PPh_3_)(SG)] + Na}^+^ adduct (overlapped with {[Au(PPh_3_)(SG)] + H + Na}^+^ in a 1.25:1.00 ratio) were found at 765.9 *m/z* (calcd. 766.1 *m/z*) and 789.1 *m/z* (calcd. 789.1 *m/z*), respectively, with the appropriate isotopic distribution pattern.

Regarding the interaction studies with cysteine (Cys), the ^1^H-NMR signals of *β* CH_2_ of the coordinated deprotonated cysteine (Cys^−^) are clearly detectable at 3.44 and 3.63 ppm. The ^31^P-NMR signal was detected at 40.6 ppm for the [Au(Cys^−^)(PPh_3_)] adduct, that is at negligibly higher field than for the [Au(PPh_3_)(SG)] adduct (see above). The C3–H ^1^H-NMR signal of the released H*2Me4Cl*aza was found at 6.36 ppm (Figure S8). Any other changes were not observed after 48 h of standing at ambient temperature, as well as in the presence of an excess (5 mol equiv) of Cys interacting with **8**.

An interaction of **8** with bovine serum albumin (BSA) resulted immediately in a white turbidity, which was subsequently removed by centrifugation (13,000 rpm for 3 min; Mini Spin, Eppendorf AG, Hamburg, Germany). The ^1^H-NMR spectrum recorded on the obtained supernatant contained the signals of the released *N*-donor ligand, with the signal positions (e.g., C3–H signal at 6.36 ppm) being consistent with those detected for free H*2Me4Cl*aza in the same mixture of solvents (Figure S8). No signals were detected in the ^31^P-NMR spectra recorded on the mentioned supernatant. On the other hand, after the centrifuged turbidity was dissolved in DMF-*d_7_*, two ^31^P-NMR signals were detected on this solution at 32.8 and 27.3 ppm, which suggested the formation two different species, including the BSA adduct with an Au–PPh_3_ residue.

### 2.4. In Vitro Antitumor Activity

The complexes **1**–**8** were studied for their in vitro cytotoxicity against two human cancer cell lines (A2780 and A2780R) and one human non-cancerous cell line (MRC-5) (Table 3).

The complexes **1**, **4**, **5,** and **8** were significantly more effective (*p* < 0.005) against the A2780 cells (IC_50_ = 2.8–3.8 μM) than conventional *cisplatin* (IC_50_ = 20.3 μM); IC_50_ stands for the half maximal inhibitory concentration. Contrary, the complexes **2**, **3**, **6**, and **7** showed only comparable in vitro anti-tumor activity with *cisplatin* (Table 3). The in vitro cytotoxicity of the most active complex **8** is more than seven times higher than *cisplatin*. The complexes **1**, **4**, **5**, and **8** were less effective against the A2780R cells than against A2780 ones and their resistance factors (RF; Table 3), defined as the ratio of IC_50_(A2780R)/IC_50_(A2780), equalled 1.2–4.6. The complexes **2**, **3**, **6**, and **7** showed comparable or even higher potency against the A2780R cells as compared with A2780 ones, resulting in the RF values of 0.6–1.1 (Table 3). All the studied complexes, except for **1**, showed moderate toxicity against the MRC-5 cells (Table 3). The values of selectivity indexes (SI = IC_50_(MRC-5)/IC_50_(A2780)) were calculated as being equal to ca. 1.2–9.6 for **1**–**8**.

### 2.5. Cell Cycle Analysis

**Figure 4:**
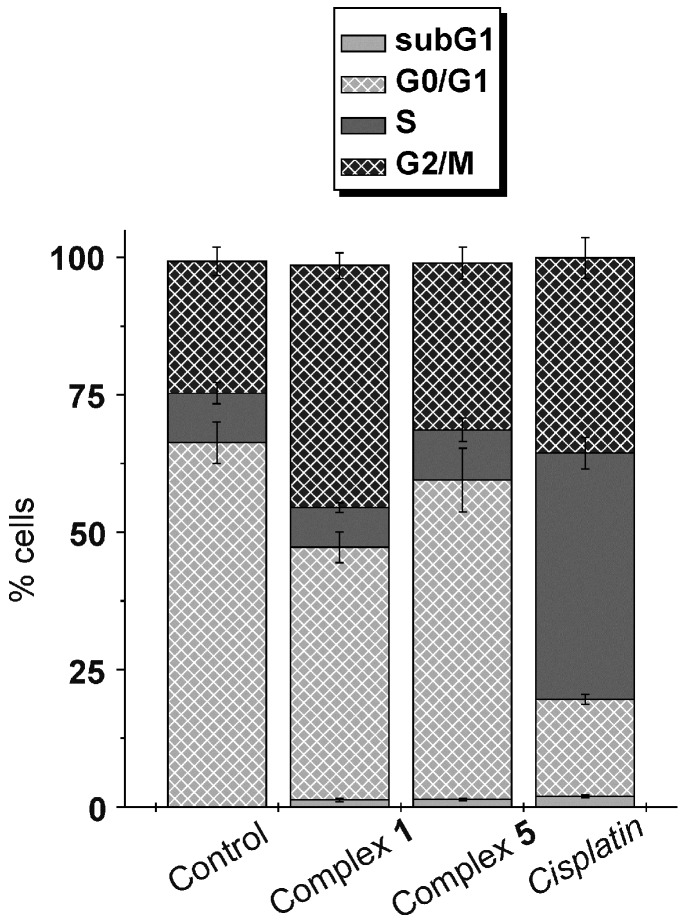
Populations in cell cycle phases studied against the A2780 human ovarian carcinoma cells treated with the complexes **1** and **5**, and *cisplatin* for comparative purposes (control = non-treated cells). Cells were stained with propidium iodide (PI)/RNase. The data are given as the arithmetic mean ± SD from three independent experiments carried out on cells from three consecutive passages.

The cell cycle modification was studied by flow cytometry (propidium iodide (PI) staining), using the A2780 cells treated by the IC_50_ concentrations of the representative complexes **1** and **5** (and *cisplatin* for comparative purposes). The obtained results are depicted in Figure 4.

Treatment of the A2780 cells by the representative complexes **1** and **5** caused a G_2_ cell cycle phase arrest (populations of 44.0% ± 2.3% for **1** and 30.4% ± 2.9% for **5**), because the % of the populations were higher than in the case of the non-treated A2780 cells (control; 24.0% ± 2.6%) (Figure 4). The G_2_-arrest was connected with a decrease of the G_0_/G_1_ cell cycle phase population of the treated A2780 cells (46.0% ± 2.8% for **1** and 58.1% ± 5.8% for **5**), as compared with control (66.3% ± 3.8%). On the other hand, the S cell cycle phase populations of the cells treated by **1** (7.2% ± 0.9%) and **5** (9.1% ± 2.1%) were comparable with the control cells (9.0% ± 1.9%), but considerably different from *cisplatin* (44.8% ± 2.9%).

## 3. Discussion

Although the complex **1** was previously reported by Chan et al. [28], we decided to use a different synthetic procedure for the syntheses of the studied triphenylphosphanegold(I) complexes **1**–**8**. The reported protocol [28] used silver(I) triflate to precipitate the chloride anions released from [AuCl(PPh_3_)] after the addition of Haza deprotonated in situ by triethylamine. In contrast to this protocol, NaOH was used in this work to deprotonate the 7-azaindoles, allowing the isolation of NaCl, which formed as a side product of the performed reactions, as previously reported in the literature (Figure 1) [19,30]. The main reason is that previously used silver(I) triflate could cause unwanted silver contamination for the below-described biological studies.

The *N*1-coordination mode of the used *n*aza ligands was proved for the crystallographically characterized complexes [Au(*3I5Br*aza)(PPh_3_)] (**7**) and [Au(*2Me4Cl*aza)(PPh_3_)]·½H_2_O (**8**′) (Figure 2). Moreover, the results of multinuclear and 2D NMR studies confirmed this coordination mode also for the studied complexes **1**–**6**, because their ^1^H-NMR and ^13^C-NMR coordination shift values (defined as ∆δ = δ_complex_–δ_ligand_; ppm) were consistent with the mentioned **7** and **8**. On the other hand, the observation of the negative ∆δ values detected for all the aromatic C–H hydrogens of the *n*aza ligands points out a different coordination mode from previously-reported platinum(II) or ruthenium(II) complexes containing the electroneutral 7-azaindole-based *N*-donor ligands coordinated through the N7 nitrogen atom [25,26,27]. The complexes were also characterized by ^31^P-NMR, however, a comparison of the ^31^P-NMR signal positions of **1**–**8** with the [AuCl(PPh_3_)] precursor was prevented by the low stability of [AuCl(PPh_3_)] in DMF-*d_7_* (two signals were detected at 33.7 and 25.6 ppm). With respect to this fact, the ^31^P-NMR spectra were recorded also for the CDCl_3_ solutions of the representative complex **8** and [AuCl(PPh_3_)], both showing one PPh_3_ signal at 33.6 ppm and 33.8 ppm, respectively, thus indicating a slight upfield shift as a consequence of the replacement of the chlorido ligand by *2Me4Cl*aza anion.

As it is known for biologically-active substituted-phosphanegold(I) complexes, such as *auranofin*, the mechanism of their action is connected with the ligand displacement [5,36,37,38]. In particular, the thiolate ligand of *auranofin* is replaced by some of the biomolecules (e.g., albumin, cysteine, or glutathione), while the *P*-donor PEt_3_ ligand of *auranofin* could be replaced (and subsequently oxidized to urine-excretable O=PEt_3_) in the excess of some of the mentioned thiols, resulting, for example, in the [Au(SG)_2_]^−^ gold-glutathione adduct [36]. A similar mechanism was also described for [AuCl(PPh_3_)], whose interaction with GSH provided the [Au(PPh_3_)(SG)] adduct, subsequently releasing its PPh_3_ ligand in the presence of GSH or 5,5′-dithiobis-(2-nitrobenzoic acid; dtnb) [15]. For these reasons, we also studied the solution chemistry and interactions with relevant biomolecules for the representative complex **8**. The studies of the solution behaviour of **8** in water-containing solution (50% DMF-*d_7_*/50% D_2_O) showed on its hydrolytic stability, because no new signals were detected in the ^1^H and ^31^P-NMR spectra even after 48 h of standing at ambient temperature. Moreover, the position of the signals detected in the ^1^H and ^31^P-NMR spectra of **8** differ from those of the starting compounds (H*2Me4Cl*aza, PPh_3_ or [AuCl(PPh_3_)]) or possible product of the decomposition of **8** (O=PPh_3_) dissolved in the same mixture of solvents (Figure 3). With respect to these results of ^1^H and ^31^P-NMR, we can disclaim that the peak of the {[Au(PPh_3_)] + O + 2H}^+^ species detected in the ESI+ mass spectra at 477.0 *m/z* (calcd. 477.1 *m/z*) resulted from the hydrolysis of **8**, but its formation is most likely connected with the coordination of water molecule to the [Au(PPh_3_)]^+^ species under the electrospray ionization conditions.

The results of the interaction experiment of **8** with GSH (1 molar equivalent) indicated an immediate covalent bonding between both the compounds, probably connected with the formation of the [Au(PPh_3_)(SG)] adduct (Figure 3). This is evidenced by the detection of the new Cys-*β* CH_2_ signals [39]; the Cys-*α* CH signal of the GS–Au adduct was not observed, because it was overlapped by the signal of water. The ^31^P-NMR signal shift is caused by the replacement of *2Me4Cl*aza by SG within a resulting [Au(PPh_3_)(SG)] adduct. The signal positions of the released *N*-donor ligand correlated well with those of free H*2Me4Cl*aza dissolved in the same mixture of solvents, as depicted in Figure 3. Interestingly, similar experiment performed with 5 mol equiv of GSH did not lead to the release of the coordinated PPh_3_ ligand and formation of a [Au(SG)_2_]^−^ adduct, because a) any new ^1^H-NMR signals were not detected for glutathione (Figure S7), and b) no ^31^P-NMR signals were detected for the released PPh_3_ molecule or its oxidized form (i.e., O=PPh_3_; Figure S6). In other words, the ^31^P-NMR spectrum of the complex **8** interacting with an excess of GSH contained only one signal with the same chemical shift as in the case of the [Au(PPh_3_)(SG)] adduct, even after 48 h of standing at ambient temperature. The results of ESI+ mass spectrometry are consistent with those of ^1^H and ^31^P-NMR spectroscopy and proved the covalent interaction of the complex **8** with GSH. Similar results were also obtained when the representative complex **8** interacted with another naturally occurring biomolecule, cysteine [40]. This interaction also resulted in the replacement of the *2Me4Cl*aza ligand by Cys (Figure S8), as proved by ^1^H and ^31^P-NMR studies. Again, a considerable downfield shift of the ^31^P-NMR signal of PPh_3_ ligand was observed as a consequence of the mentioned changes within the inner coordination sphere. This phenomenon is known from the literature, where the results of the ^31^P-NMR study performed on the [Au(L)(PPh_3_)] complexes containing different *N*-donor ligand (L = 4-picoline, 2-amine-4-picoline or *N*,*N*-dimethylaminopyridine) interacting with *N*-acetyl-l-cysteine (1 mol equiv) in CD_3_CN showed similar downfield shift from 30 to ca. 38 ppm [41]. The results of the interaction studies with BSA indicated that the used biomolecule also replaces the *N*-donor ligand *2Me4Cl*aza in the structure of the representative complex **8** (Figure S8), resulting in the Au–BSA adduct insoluble in the used mixture of solvents (50% DMF-*d_7_*/50% D_2_O).

The results of the in vitro cytotoxicity testing indicated different potency of **1**, **4**, **5**, and **8** (significantly more potent than *cisplatin*), and **2**, **3**, **6**, and **7** (comparable potency with *cisplatin*) against the A2780 cells (Table 3). Interestingly, although the A2780 cells are naturally sensitive to the biological action of various types of gold(I) complexes including the substituted-phosphane ones (low micromolar or even nanomolar range of the IC_50_ values), the potency of the previously reported substituted-phosphanegold(I) complexes is usually comparable or only slightly higher than *cisplatin* [18,21,22,42,43,44,45]. In other words, the seven-fold higher in vitro anticancer activity of **8** against the A2780 cells seems to be, to the best of our knowledge, exceptional in the field of gold(I) complexes. Regarding *auranofin*, it showed ca. three-fold higher potency in vitro against the A2780 cells than *cisplatin* [42]. The complexes **1**–**8** were also tested against the A2780R cancer cell line with acquired resistance to *cisplatin*. The obtained results, calculated as the resistance factors (RF; Table 3), are suggestive for the ability of **2**, **3**, **6**, and **7** to overcome the acquired resistance of the used human ovarian carcinoma cells to the therapeutic action of *cisplatin*, which is comparable with *auranofin* (RF = ca. 0.9) [42] or some other gold(I) complexes reported as effectively circumventing the acquired resistance of the A2780 cells to *cisplatin* [18,45]. Moderate toxicity of the complexes **4**, **5**, and **8** against the MRC-5 normal human fibroblast cells resulted, together with their high cytotoxicity against the A2780 cells, in the promising values (8.3–9.6) of selectivity indexes (SI; Table 3). Selectivity is generally accepted in the field of anticancer potent gold complexes as one of the main advantage of these substances over the conventional platinum-based drugs. For example triphenylphosphanegold(I) complexes, structurally similar to the complexes **1**–**8**, which contain 9-deazahypoxanthines as *N*-donor ligand, were reported as compounds showing selective potency towards the cancer cells (especially human MCF-7 breast carcinoma and HOS osteosarcoma cells with SIs up to 30) over the normal cells (human hepatocytes were used) [22]. However, their SI values at the A2780 cells (ca. 4.6–5.2) are lower than for the herein studied complexes **4**, **5**, and **8**. Regarding *auranofin*, its selectivity was very recently reported as comparable with chlorido-triphenylphosphanegold(I) complex and, more importantly, with *cisplatin*, as resulted from the studies using the B16–F10 metastatic skin melanoma, CT26-WT colon cancer, and 4T1 mammary adenocarcinoma cancer cells and their comparison with non-cancerous BHK21 kidney cells [46].

Both the studied complexes **1** and **5** showed similar trends, but with a different extent of the A2780 cancer cell cycle perturbation (Figure 4). In particular, treatment by the complexes **1** and **5** led to a G_2_-arrest, because their populations were higher than those of the non-treated (control) cells. Consequently, the G_0_/G_1_ population decreased for **1** and **5** as compared with the control cells, while the number of cells in the S cell cycle phase was comparable for **1** and **5**, and the non-treated control cells. Different modification of the A2780 cell cycle, induced by the studied gold(I) complexes (i.e., G_2_-arrest) and *cisplatin* (i.e., S-arrest), is indicative for different mechanism of anticancer action of **1** and **5**, as compared with conventional *cisplatin* [47]. The mentioned G_2_-arrest observed at the A2780 cells treated by the complexes **1** and **5** is different from the cell cycle modification provoked by different gold(I) complexes including *auranofin*. In particular, *auranofin* induced apoptosis connected with the sub-G_1_ cell cycle phase population increase (>13% as compared with untreated cells) in the A549 human lung carcinoma cells [48]. Similar increase of the sub-G_1_ population was reported for different triethylphosphanegold(I) complexes containing CN^−^, SCN^−^ or xanthogenate anion counter-ligand using the 2008 human ovarian carcinoma cells [49]. It is of interest, that *auranofin*, an apoptosis-inducer at the A549 cells (see above), caused the G_0_/G_1_ cell cycle arrest when it was used for the treatment of the U266 human myeloma cells [50]. The [Au(L)(PPh_3_)] complexes (L = 4-picoline, 2-amine-4-picoline or *N*,*N*-dimethylaminopyridine), structurally similar to the herein studied complexes **1**–**8**, at the HCT116 human colon carcinoma cells, as well as gold(I) complex of the bis-chelated gold(I)-diphosphane type at the A549 cancer cells, induced the similar increase of the G_0_/G_1_ cell cycle population [19,44].

Taken together, the treatment of the A2780 cells by the complexes **1** and **5** led the cell cycle to different perturbations, compared to those recently reported for other gold(I) complexes (including *auranofin*) as well as for *cisplatin*. Thus, this observation has to be taken into account as suggestive for a different mechanism of action as compared with the above-named substances.

## 4. Materials and Methods

### 4.1. Materials

H[AuCl_4_]∙3H_2_O, triphenylphosphane (PPh_3_), 7-azaindole (Haza), 3-chloro-7-azaindole (H*3Cl*aza), 3-bromo-7-azaindole (H*3Br*aza), 3-iodo-7-azaindole (H*3I*aza), 5-bromo-7-azaindole (H*5Br*aza), 3-chloro-5-bromo-7-azaindole (H*3Cl5Br*aza), 3-iodo-5-bromo-7-azaindole (H*3I5Br*aza), 2-methyl-4-chloro-7-azaindole (H*2Me4Cl*aza), NaOH, *cisplatin*, reduced glutathione (GSH), cysteine, BSA, Roswell Park Memorial Institute medium, fetal calf serum, glutamine, penicillin and streptomycin, and solvents (acetone, methanol, diethyl ether, chloroform, DMF, DMSO) and solvents for NMR experiments (DMF-*d_7_*, D_2_O) were supplied by Sigma-Aldrich Co. (Prague, Czech Republic) and Fisher-Scientific Co. (Pardubice, Czech Republic).

The starting chlorido-triphenylphosphanegold(I) complex, [AuCl(PPh_3_)], was prepared as described previously [51], and its composition was checked by elemental analysis and FTIR spectroscopy.

### 4.2. Synthesis

One mol of NaOH (0.6 mL) was poured into the mixture of [AuCl(PPh_3_)] (0.5 mmol) and the appropriate 7-azaindole-based ligand (0.6 mmol) in acetone (10 mL). After 48 h of stirring at 50 °C, the obtained mixtures, containing the white solid, were filtered. The volume of the clear colourless filtrates was reduced until the off-white precipitate formed (**1**–**4**, **6** and **8**). In the case of the complexes **5** and **7**, the brown gel was obtained when the solvent was removed, which crystalized to the off-white precipitate under diethyl ether. The products (Figure 1) were collected by filtration, washed with diethyl ether (3 × 5 mL), and dried under vacuum.

[Au(aza)(PPh_3_)] (**1**): *Anal.* Calcd. for C_25_H_20_N_2_PAu: C, 51.7; H, 3.6; N, 4.8; Found: C, 51.5; H, 3.6; N, 4.4%. ^1^H-NMR (DMF-*d_7_*, 400 MHz, 300 K): 8.14 (1H, *m*, C6–H), 7.90 (1H, *m*, C4–H), 7.73 (15H, *m*, PPh_3_), 7.52 (1H, *d*, *J* = 3.1 Hz, C2–H), 6.89 (1H, *m*, C5–H), 6.38 (1H, *d*, *J* = 3.1 Hz, C3–H) ppm. ^13^C-NMR (DMF-*d_7_*, 100 MHz, 300 K): 160.1 (C7a), 141.6 (C6), 137.7 (C2), 135.2, 135.1, 130.7, 130.6, 129.7 (PPh_3_), 127.7 (C4), 122.2 (C3a), 114.8 (C5), 99.8 (C3) ppm. ^31^P-NMR (DMF-*d_7_*, 243 MHz, 300 K): 32.7 (P1) ppm.

[Au(*3Cl*aza)(PPh_3_)] (**2**): *Anal.* Calcd. for C_25_H_19_N_2_ClPAu: C, 49.2; H, 3.1; N, 4.6; Found: C, 48.9; H, 3.1; N, 4.5%. ^1^H-NMR (DMF-*d_7_*, 400 MHz, 300 K): 8.24 (1H, *dd*, *J* = 4.6, 1.5 Hz, C6–H), 7.89 (1H, *dd*, *J* = 7.9, 1.3 Hz, C4–H), 7.75 (15H, *m*, PPh_3_), 7.63 (1H, *s*, C2–H), 7.03 (1H, *m*, C5–H) ppm. ^13^C-NMR (DMF-*d_7_*, 100 MHz, 300 K): 155.6 (C7a), 143.0 (C6), 135.4, 135.2 (PPh_3_), 133.3 (C2), 130.8, 130.6, 130.3, 129.7 (PPh_3_), 125.4 (C4), 119.5 (C3a), 115.6 (C5), 101.2 (C3) ppm. ^31^P-NMR (DMF-*d_7_*, 243 MHz, 300 K): 32.9 (P1) ppm.

[Au(*3Br*aza)(PPh_3_)] (**3**): *Anal.* Calcd. for C_25_H_19_N_2_BrPAu: C, 45.5; H, 3.0; N, 4.2; Found: C, 45.0; H, 2.9; N, 4.0%. ^1^H-NMR (DMF-*d_7_*, 400 MHz, 300 K): 8.23 (1H, *m*, C6–H), 7.83 (1H, *m*, C4–H), 7.74 (15H, *m*, PPh_3_), 7.65 (1H, *s*, C2–H), 7.04 (1H, *m*, C5–H) ppm. ^13^C-NMR (DMF-*d_7_*, 100 MHz, 300 K): 156.2 (C7a), 143.2 (C6), 136.9 (C7), 135.4, 135.3 (PPh_3_), 133.4 (C2), 130.8, 130.7, 130.4, 129.9 (PPh_3_), 126.2 (C4), 121.1 (C3a), 115.7 (C5), 86.3 (C3) ppm. ^31^P-NMR (DMF-*d_7_*, 243 MHz, 300 K): 32.8 (P1) ppm.

[Au(*3I*aza)(PPh_3_)] (**4**): *Anal.* Calcd. for C_25_H_19_N_2_IPAu: C, 42.8; H, 2.7; N, 4.0; Found: C, 42.7; H, 2.4; N, 4.1%. ^1^H-NMR (DMF-*d_7_*, 400 MHz, 300 K): 8.20 (1H, *d*, *J* = 4.4 Hz, C6–H), 7.72 (17H, *m*, C4–H, C2–H, PPh_3_), 7.03 (1H, *dd*, *J* = 7.5, 4.9 Hz, C5–H) ppm. ^13^C-NMR (DMF-*d_7_*, 100 MHz, 300 K): 156.8 (C7a), 143.0 (C6), 142.0 (C2), 135.1, 130.7, 130.6, 130.1, 129.7 (PPh_3_), 127.7 (C4), 124.2 (C3a), 116.0 (C5), 54.6 (C3) ppm. ^31^P-NMR (DMF-*d_7_*, 243 MHz, 300 K): 32.8 (P1) ppm.

[Au(*5Br*aza)(PPh_3_)] (**5**): *Anal.* Calcd. for C_25_H_19_N_2_BrPAu: C, 45.8; H, 2.9; N, 4.3; Found: C, 45.6; H, 2.8; N, 4.0%. ^1^H-NMR (DMF-*d_7_*, 400 MHz, 300 K): 8.10 (1H, *s*, C6–H), 8.10 (1H, *s*, C4–H), 7.71 (1H, *s*, C2–H), 7.70 (15H, *m*, PPh_3_), 6.65 (1H, *d*, *J* = 2.8 Hz, C3–H) ppm. ^13^C-NMR (DMF-*d_7_*, 100 MHz, 300 K): 156.0 (C7a), 141.6 (C6), 139.9 (C2), 135.4, 135.2 (PPh_3_), 133.7 (C4), 130.8, 130.7, 129.7 (PPh_3_), 124.3 (C3a), 110.1 (C5), 99.9 (C3) ppm. ^15^N-NMR (DMF-*d_7_*, 40 MHz, 300 K): 276.7 (N7), 188.9 (N1) ppm. ^31^P-NMR (DMF-*d_7_*, 243 MHz, 300 K): 33.2 (P1) ppm.

[Au(*3Cl5Br*aza)(PPh_3_)] (**6**): *Anal.* Calcd. for C_25_H_18_N_2_BrClPAu: C, 43.5; H, 2.6; N, 4.1; Found: C, 43.2; H, 2.6; N, 3.9%. ^1^H-NMR (DMF-*d_7_*, 400 MHz, 300 K): 8.26 (1H, *s*, C6–H), 8.02 (1H, *s*, C4–H), 7.72 (15H, *m*, PPh_3_), 7.72 (1H, *s*, C2–H) ppm. ^13^C-NMR (DMF-*d_7_*, 100 MHz, 300 K): 154.0 (C7a), 143.2 (C6), 135.4, 135.3 (PPh_3_), 133.4 (C2), 130.8, 130.6, 130.2, 129.8 (PPh_3_), 127.4 (C4), 121.1 (C3a), 110.8 (C5), 101.0 (C3) ppm. ^31^P-NMR (DMF-*d_7_*, 243 MHz, 300 K): 33.1 (P1) ppm.

[Au(*3I5Br*aza)(PPh_3_)] (**7**): *Anal.* Calcd. for C_25_H_18_N_2_BrIPAu: C, 38.3; H, 2.4; N, 3.6; Found: C, 38.1; H, 2.1; N, 3.8%. ^1^H-NMR (DMF-*d_7_*, 400 MHz, 300 K): 8.23 (1H, *d*, *J* = 2.2 Hz, C6–H), 7.73 (17H, *m*, C4–H, C2–H, PPh_3_) ppm. ^13^C-NMR (DMF-*d_7_*, 100 MHz, 300 K): 152.2 (C7a), 144.2 (C6), 143.10 (C2), 135.3, 135.1 (PPh_3_), 132.8 (C4), 130.8, 130.7, 130.2, 129.8 (PPh_3_), 126.0 (C3a), 111.1 (C5), 55.6 (C3) ppm. ^31^P-NMR (DMF-*d_7_*, 243 MHz, 300 K): 32.8 (P1) ppm.

[Au(*2Me4Cl*aza)(PPh_3_)] (**8**): *Anal.* Calcd. for C_25_H_19_N_2_BrPAu: C, 50.0; H, 3.4; N, 4.5; Found: C, 49.7; H, 3.3; N, 4.4%. ^1^H-NMR (DMF-*d_7_*, 400 MHz, 300 K): 7.97 (1H, *m*, C6–H), 7.74 (15H, *m*, PPh_3_), 6.93 (1H, *m*, C5–H), 6.19 (1H, *s*, C3–H), 2.59 (3H, *s*, CH_3_) ppm. ^13^C-NMR (DMF-*d_7_*, 100 MHz, 300 K): 158.9 (C7a), 148.0 (C2), 141.0 (C6), 135.4, 135.3 (PPh_3_), 133.4 (C4), 130.8, 130.7, 130.4, 130.0 (PPh_3_), 122.3 (C3a), 114.3 (C5), 96.7 (C3), 18.1 (CH_3_) ppm. ^15^N-NMR (DMF-*d_7_*, 40 MHz, 300 K): 269.2 (N7), 203.3 (N1) ppm. ^31^P-NMR (DMF-*d_7_*, 243 MHz, 300 K): 33.1 (P1) ppm.

### 4.3. General Methods

Elemental analysis was carried out using a Flash 2000 CHNS Elemental Analyzer (Thermo Scientific, Waltham, MA, USA). ^1^H and ^13^C-NMR spectroscopy, and ^1^H–^1^H gsCOSY, ^1^H–^13^C gsHMQC and ^1^H–^13^C gsHMBC two-dimensional correlation experiments of the DMF-*d_7_* or CDCl_3_ solutions of **1**–**8** were performed at 300 K on a Varian 400 device (at 400.0 MHz (^1^H) or 100.6 MHz (^13^C)); gs = gradient selected, COSY = correlation spectroscopy, HMQC = heteronuclear multiple quantum coherence, HMBC = heteronuclear multiple bond coherence. ^1^H and ^13^C-NMR spectra were calibrated against the residual DMF ^1^H-NMR (8.03, 2.92 and 2.75 ppm) and ^13^C-NMR (163.2, 34.9 and 29.8 ppm) signals. The splitting of proton resonances in the reported ^1^H spectra is defined as s = singlet, d = doublet, dd = doublet of doublets and m = multiplet. ^1^H–^15^N gsHMBC experiment was carried out at natural abundance for the complex **5** and calibrated against the residual signals of DMF adjusted to 8.03 ppm (^1^H) and 104.7 (^15^N) ppm. ^31^P-NMR spectra were recorded for the complex **8** (and [AuCl(PPh_3_)]) and calibrated against 85% H_3_PO_4_ used as an internal reference standard. Electrospray ionization mass spectrometry (ESI-MS) was performed by LCQ Fleet ion trap spectrometer (Thermo Scientific; QualBrowser software, version 2.0.7) in both the positive (ESI+) and negative (ESI−) ionization modes on the methanol solutions. FTIR spectra were recorded by a Nexus 670 FT-IR spectrometer (Thermo Nicolet, Waltham, MA, USA) at 150–600 cm^−1^ (far-IR) and 400–4000 cm^−1^ (mid-IR), using the ATR technique. Raman spectroscopy was performed on a NXR FT-Raman Module (Thermo Nicolet, Waltham, MA, USA) between 250 and 3700 cm^−1^.

### 4.4. Single Crystal X-ray Analysis

The crystals of the complexes [Au(*3I5Br*aza)(PPh_3_)] (**7**) and [Au(*2Me4Cl*aza)(PPh_3_)]·½H_2_O (**8**′) suitable for a single crystal X-ray analysis were prepared by slow evaporation of the solutions of the named complexes in the mixture of dichloromethane and *n*-hexane (1:1, *v/v*). X-ray data of both crystals were collected on a Bruker (Billerica, MA, USA) D8 QUEST diffractometer equipped with a PHOTON 100 CMOS detector (Bruker) using Mo–K_α_ radiation. The APEX3 software package was used for data collection and reduction [52]. The structures were solved using a direct method and refined using the Bruker SHELXTL Software Package [53]. The graphics were drawn and additional structural calculations were performed using DIAMOND [54] and Mercury [55] software.

### 4.5. Studies of Aqueous Chemistry and Interaction with Biomolecules

Complex **8** and [AuCl(PPh_3_)] (for comparative purposes) were dissolved in 600 µL of the 50% DMF-*d_7_*/50% D_2_O mixture to give the solutions of ca. 1 mM concentration; a presence of DMF ensured the solubility of the complex **8**, with respect to its low solubility in water. Similar solutions were prepared with **1** or **5** mol equivs of GSH, Cys, or BSA. ^1^H and ^31^P-NMR spectra were recorded at different time points over 48 h at 300 K. The obtained ^1^H-NMR spectra were calibrated against the residual signal of water found at 4.75 ppm, while the ^31^P-NMR spectra were referenced as described above.

Similar experiments were carried out also in non-deuterated solvents and evaluated by ESI+ mass spectrometry.

### 4.6. In Vitro Cytotoxicity Testing

Human A2780 ovarian carcinoma, A2780R *cisplatin*-resistant ovarian carcinoma and MRC-5 normal fibroblast cell lines were supplied by the European Collection of Cell Cultures (ECACC). The cell lines were cultured, according to the supplier´s instructions, in RPMI-1640 medium supplemented with 10% of fetal calf serum, 1% of 2 mM glutamine, and 1% penicillin/streptomycin. All cell lines were grown as adherent monolayers at 37 °C and 5% CO_2_ in a humidified atmosphere.

An appropriate amount of the complexes **1**–**8** (or *cisplatin* involved in the study as standard) was dissolved in DMF to give the 50 mM stock solutions. The cells were seeded to 96-well culture plates, pre-incubated in drug-free medium at 37 °C for 24 h and treated with the 0.01–10.0 μM solutions (for the complex **1** being less soluble in the used medium) or 0.01–50.0 μM solutions (for sufficiently soluble complexes **2**–**8** and *cisplatin*) prepared from the stock solutions by dilution with medium, for 24 h at 37 °C. In parallel, the cells were also treated with vehicle (0.1% DMF in medium; negative control) and Triton X-100 (1%; positive control) to assess the minimal and maximal cell damage, respectively. The MTT assay was used to determine the cell viability. A concentration of the formed dye was evaluated spectrophotometrically at 540 nm (TECAN, Schoeller Instruments LLC, Brno, Czech Republic). The data were expressed as the percentage of viability, where 100% and 0% represent the treatments with negative and positive controls, respectively. The data were acquired from three independent experiments (conducted in triplicate) using cells from different passages. The resulting IC_50_ values (µM) were calculated from viability curves and the results are presented as the arithmetic mean ± SD.

### 4.7. Cell Cycle Analysis

A2780 cells (1.0 × 10^6^ per well) were pre-incubated in a six-well plate for 24 h, as described above. The selected complexes **1** and **5** were added at the concentrations equal to their IC_50_ (*cisplatin* was involved in the study for comparative purposes). After 24 h, floating cells were collected and attached cells were harvested using trypsin/EDTA in PBS. Total cells were washed twice with PBS and fixed in 70% ethanol. Cells were resuspended in PBS and DNA staining was achieved by a solution of propidium iodide (PI) supplemented with RNase A (30 min, 25 °C, in the dark). After that, the cells were washed (PBS), resuspended (PBS), and DNA content was measured using flow cytometry (CytoFlex, Beckman Coulter, Brea, CA, USA) detecting emission of DNA-bound PI (maximum at 617 nm) after excitation at 535 nm. The data were analysed using CytExpert^TM^ software (Beckman Coulter).

### 4.8. Statistical Analysis

An analysis of variance (ANOVA) test was used for statistical analysis with the values of *p* < 0.05 (*), 0.01 (**) and 0.005 (***) considered to be statistically significant. QC Expert 3.2 Statistical software (TriloByte Ltd., Crewe, UK) was used to perform the analysis.

## 5. Conclusions

In the present work, eight triphenylphosphanegold(I) complexes containing the deprotonated 7-azaindole or its variously substituted derivatives (*n*aza) were investigated. Complexes [Au(*3I5Br*aza)(PPh_3_)] (**7**) and [Au(*2Me4Cl*aza)(PPh_3_)]·½H_2_O (**8**′) were crystallographically characterized, showing the used 7-azaindole derivatives as coordinated through their N1 atoms. The complexes **4**, **5**, and **8** exhibited low-micromolar in vitro anticancer potency against the A2780 cells (IC_50_ = 2.8–3.5 µM; 24 h exposure time), which is significantly higher than that of *cisplatin*. These complexes were markedly less effective (IC_50_ = 26.0–29.2 µM) against the MRC-5 cells, which is suggestive for promising selectivity towards cancer cells over the normal ones. The treating of the A2780 cells by the representative complexes **1** and **5** (IC_50_ concentrations were used) led to different cell cycle modification (G2-arrest) as compared with the cells treated by the conventional platinum-based anticancer drug *cisplatin*. The representative complex **8** was hydrolytically stable in the water-containing solution (50% DMF-*d_7_*/50% D_2_O mixture), while it released its *N*-donor ligand when mixed with relevant naturally occurring biomolecules, i.e., reduced glutathione, cysteine, or bovine serum albumin, as proved by the detailed ^1^H and ^31^P-NMR, and ESI+ MS studies.

## Figures and Tables

**Table 1:** Crystal data and structure refinements for the complexes [Au(*3I5Br*aza)(PPh_3_)] (**7**) and [Au(*2Me4Cl*aza)(PPh_3_)]·½H_2_O (**8**′).

	7	8′
Empirical Formula	C_25_H_18_AuBrIN_2_P	C_52_H_44_Au_2_Cl_2_N_4_OP_2_
Formula weight	781.16	1267.68
Temperature (K)	120(2)	120(2)
Wavelength (Å)	0.71073	0.71073
Crystal system, space group	Monoclinic, *P*2_1_/c	Monoclinic, *C*2/c
Unit cell dimensions		
*a* (Å)	6.8563(3)	24.2844(14)
*b* (Å)	24.2482(12)	15.1186(14)
*c* (Å)	14.7081(8)	16.3330(14)
*α* (°)	90	90
*β* (°)	101.313(2)	128.203(4)
*γ* (°)	90	90
*V* (Å^3^)	2397.8(2)	4712.3(7)
*Z*, *D_calc_* (g·cm^−3^)	4, 2.164	4, 1.787
Absorption coefficient (mm^−1^)	9.172	6.444
Crystal size (mm)	0.170 × 0.140 × 0.120	0.160 × 0.120 × 0.120
*F* (000)	1456	2456
*θ* range for data collection (°)	2.825 to 27.513	2.500 to 27.940
Index ranges (*h*, *k*, *l*)	−7 ≤ *h* ≤ 8	−31 ≤ *h* ≤ 31
	−31 ≤ *k* ≤ 31	−19 ≤ *k* ≤ 19
	−19 ≤ *l* ≤ 19	−21 ≤ *l* ≤ 21
Reflections collected/unique	56041	104819
Data/restraints/parameters	5508/0/280	5648/0/289
Goodness–of–fit on *F*^2^	1.071	1.012
Final *R* indices [*I* > 2*σ*(*I*)]	*R*_1_ = 0.0279, w*R*_2_ = 0.0453	*R*_1_ = 0.0193, w*R*_2_ = 0.0340
*R* indices (all data)	*R*_1_ = 0.0427, w*R*_2_ = 0.0485	*R*_1_ = 0.0301, w*R*_2_ = 0.0366
Largest peak and hole (e Å^−3^)	0.900 and −1.237	0.550 and −0.702
Cambridge Crystallographic Data Centre nos.	1507789	1507790

**Table 2:** Selected bond lengths (Å) and angles (°) for the complexes [Au(*3I5Br*aza)(PPh_3_)] (**7**) and [Au(*2Me4Cl*aza)(PPh_3_)]·½H_2_O (**8**′).

Parameter	7	8′
Au1–N1	2.047(3)	2.030(2)
Au1–P1	2.2349(9)	2.2277(6)
P1–Au1–N1	176.41(8)	173.27(6)
Au1–P1–C10	112.34(12)	107.47(8)
Au1–P1–C20	111.24(12)	117.05(8)
Au1–P1–C30	115.36(11)	114.19(8)
Au1–N1–C2	126.1(2)	125.1(2)
Au1–N1–C7a	125.9(2)	127.7(2)

**Table 3:** The results of the in vitro cytotoxicity studies of gold(I) complexes **1**–**8** (and *cisplatin* for comparative purposes) against the human A2780 ovarian carcinoma, A2780R *cisplatin*-resistant ovarian carcinoma and MRC-5 normal fibroblast cell lines, performed with the 24 h exposure time. Data are expressed as half maximal inhibitory concentration (IC_50_) ± standard deviation (SD) (μM). The significant differences between the IC_50_ values for **1**–**8** and *cisplatin* are given as *** *p* < 0.005.

Complex	A2780	A2780R	MRC5	RF ^1^	SI ^2^
[Au(aza)(PPh_3_)] (1)	3.8 ± 1.1 ***	4.4 ± 0.8	7.7 ± 1.7	1.2	2.0
[Au(*3Cl*aza)(PPh_3_)] (2)	22.4 ± 5.7	21.7 ± 0.8	31.4 ± 4.9	1.0	1.4
[Au(*3Br*aza)(PPh_3_)] (3)	23.3 ± 3.4	21.3 ± 0.8	27.3 ± 1.7	0.9	1.2
[Au(*3I*aza)(PPh_3_)] (4)	3.5 ± 0.4 ***	11.0 ± 4.7	29.2 ± 5.9	3.1	8.3
[Au(*5Br*aza)(PPh_3_)] (5)	3.1 ± 0.5 ***	14.4 ± 3.3	26.0 ± 4.2	4.6	8.4
[Au(*3Cl5Br*aza)(PPh_3_)] (6)	22.9 ± 2.3	13.8 ± 2.1	26.5 ± 3.7	0.6	1.2
[Au(*3I5Br*aza)(PPh_3_)] (7)	20.2 ± 2.5	22.1 ± 0.3	27.3 ± 2.6	1.1	1.4
[Au(*2Me4Cl*aza)(PPh_3_)] (8)	2.8 ± 0.7 ***	8.9 ± 3.8	26.9 ± 3.6	3.2	9.6
*Cisplatin*	20.3 ± 2.3	>50.0	>50.0	-	-

^1^ RF = resistance factor (calculated as IC_50_(A2780R)/IC_50_(A2780)); ^2^ SI = selectivity index (calculated as IC_50_(MRC5)/IC_50_(A2780)).

## Supplementary Materials

The following are available online at www.mdpi.com/1422-0067/17/12/2084/s1.
