# Supplementary material for: In Vitro Antitumor Active Gold(I) Triphenylphosphane Complexes Containing 7-Azaindoles
Source: Int J Mol Sci. 2016 Dec 11;17(12):2084. doi: 10.3390/ijms17122084 (PMC5187884; doi:10.3390/ijms17122084)
Supplement: Supplementary file 1 [file ijms-17-02084-s001.pdf]

Supplementary Materials: In Vitro Antitumor Active Gold(I) Triphenylphosphane Complexes Containing 7-Azaindoles

Pavel Štarha, Zdeněk Trávníček, Bohuslav Drahoš and Zdeněk Dvořák

1. Results of ESI Mass Spectrometry and FTIR Spectroscopy

1: ESI+ MS (methanol, *m*/*z*): 721.2 (calc. 721.2; 100%; $[Au(PPh_3)_2]^+$), 577.2 (calc. 577.1; 2%; $\{[Au(aza)(PPh_3)] + H\}^+$), 459.2 (calc. 459.1; 2%; $[Au(PPh_3)]^+$), 119.1 (calc. 119.1; 2%; $\{(Haza) + H\}^+$). ESI− MS (methanol, *m*/*z*): 431.2 (calc. 431.1; 50%; $[Au(aza)_2]^-$), 117.1 (calc. 117.0; 100%; $(aza)^-$). FTIR (ν_{ATR}/cm^{-1}): 434m, 462m, 498vs, 542vs, 617m, 639m, 690vs, 711s, 744vs, 774s, 793m, 851w, 895m, 931w, 949m, 987m, 1026m, 1046m, 1071m, 1099vs, 1169s, 1199m, 1265s, 1291s, 1312m, 1334m, 1351s, 1354s, 1404vs, 1433vs, 1463s, 1478m, 1555m, 1585s, 2918m, 2988m, 3007m, 3032m, 3055m, 3088m. FTIR (ν_{ATR}/cm^{-1}): 241w, 328w, 434m, 462m, 499vs, 543vs, 592w. Raman (cm^{-1}): 329w, 464w, 566w, 617w, 639w, 695w, 712w, 763m, 851w, 877w, 923w, 999vs, 1028m, 1042m, 1072m, 1101m, 1160w, 1185w, 1293w, 1351w, 1407w, 1438w, 1465m, 482m, 1556w, 1585s, 2954w, 3008w, 3056vs, 3108w, 3142w, 3168w.

2: ESI+ MS (methanol, *m*/*z*): 721.2 (calc. 721.2; 100%; $[Au(PPh_3)_2]^+$), 611.1 (calc. 611.1; 2%; $\{[Au(3Claza)(PPh_3)] + H\}^+$), 459.2 (calc. 459.1; 2%; $[Au(PPh_3)]^+$). ESI− MS (methanol, *m*/*z*): 499.1 (calc. 499.0; 70%; $[Au(3Claza)_2]^-$), 151.1 (calc. 151.0; 60%; $(3Claza)^-$). FTIR (ν_{ATR}/cm^{-1}): 452m, 496vs, 545s, 592m, 669vs, 712s, 747vs, 765vs, 786w, 834m, 850w, 921m, 948w, 972m, 1004s, 1027vs, 1072m, 1102vs, 1150m, 1180s, 1192s, 1204m, 1269s, 1297vs, 1324s, 1350m, 1398vs, 1434vs, 1479vs, 1545m, 1574m, 1590s, 2879m, 2914m, 3033s, 3049s, 3113m. FTIR (ν_{ATR}/cm^{-1}): 202w, 231w, 248w, 330w, 397w, 431m, 451m, 498vs, 507s, 536vs, 544vs, 591m. Raman (cm^{-1}): 200w, 435w, 542w, 569w, 617w, 694w, 713w, 764m, 923w, 999vs, 1029m, 1103m, 1159w, 1185w, 1270w, 1299w, 1326w, 1345w, 1400w, 1436w, 1480s, 1545w, 1586s, 3954w, 3053vs, 3112w, 3142w, 3171w.

3: ESI+ MS (methanol, *m*/*z*): 721.4 (calc. 721.2; 100%; $[Au(PPh_3)_2]^+$), 655.2 (calc. 655.0; 5%; $\{[Au(3Braza)(PPh_3)] + H\}^+$), 459.3 (calc. 459.1; 5%; $[Au(PPh_3)]^+$), 197.1 (calc. 197.0; 3%; $\{(H3Braza) + H\}^+$). ESI− MS (methanol, *m*/*z*): 589.2 (calc. 588.9; 100%; $[Au(3Braza)_2]^-$), 195.2 (calc. 195.0; 20%; $(3Braza)^-$). FTIR (ν_{ATR}/cm^{-1}): 435w, 450m, 498s, 524m, 542s, 588w, 617w, 688vs, 711m, 747s, 765s, 835m, 866w, 919m, 986s, 1027w, 1071w, 1103vs, 1144m, 1160w, 1181w, 1198m, 1264m, 1293s, 1320m, 1342w, 1392vs, 1433vs, 1468s, 1539w, 1585m, 1670w, 2784w, 2844w, 3029m, 3045m, 3107m, 3169w. FTIR (ν_{ATR}/cm^{-1}): 354w, 435m, 451m, 497vs, 525s, 543vs, 588m. Raman (cm^{-1}): 282w, 319w, 357w, 398w, 433w, 569w, 619w,678w, 694w, 714w, 764m, 923w, 1000vs, 1031m, 1104m, 1160w, 1185w, 1260w, 1185w, 1268w, 1297w, 1345w, 1374w, 1396w, 1437w, 1473m, 1542w, 1587s, 2955w, 2993w, 3053vs, 3110w, 3143w, 3172w.

4: ESI+ MS (methanol, *m*/*z*): 721.4 (calc. 721.2; 100%; $[Au(PPh_3)_2]^+$), 703.3 (calc. 703.0; 10%; $\{[Au(3Iaza)(PPh_3)] + H\}^+$), 459.3 (calc. 459.1; 5%; $[Au(PPh_3)]^+$), 245.1 (calc. 245.0; 5%; $\{(H3Iaza) + H\}^+$). ESI− MS (methanol, *m*/*z*): 683.0 (calc. 682.8; 100%; $[Au(3Iaza)_2]^-$), 243.1 (calc. 242.9; 80%; $(3Iaza)^-$). FTIR (ν_{ATR}/cm^{-1}): 448w, 499vs, 516m, 542vs, 587w, 616w, 689vs, 711m, 746s, 766s, 784w, 837w, 917w, 975m, 995m, 1027w, 1102vs, 1158w, 1180m, 1197m, 1215m, 1263m, 1289s, 1315m, 1332w, 1388vs, 1433vs, 1460m, 1479m, 1536w, 1552w, 1583m, 2663w, 3028m, 3047m. FTIR (ν_{ATR}/cm^{-1}): 243w, 303w, 322w, 397w, 435m, 449m, 497vs, 517s, 542vs, 586m. Raman (cm^{-1}): 280w, 303w, 325w, 520w, 543w, 567w, 617w, 668w, 693w, 712w, 762m, 919w, 999vs, 1032m, 1102m, 1158w, 1183w, 1265w, 1292w, 1318w, 1340w, 1391w, 1462m, 1543w, 1586s, 2988w, 3033m, 3050vs, 3143w, 3170w.

5: ESI+ MS (methanol, *m*/*z*): 721.4 (calc. 721.2; 100%; $[Au(PPh_3)_2]^+$), 655.2 (calc. 655.0; 10%; $\{[Au(5Braza)(PPh_3)] + H\}^+$), 459.3 (calc. 459.1; 10%; $[Au(PPh_3)]^+$), 197.1 (calc. 197.0; 2%; $\{(H5Braza) + H\}^+$). ESI− MS (methanol, *m*/*z*): 589.2 (calc. 588.9; 100%; $[Au(5Braza)_2]^-$), 195.2 (calc. 195.0; 70%; $(5Braza)^-$). FTIR (ν_{ATR}/cm^{-1}): 432w, 471m, 499s, 541vs, 609m, 689vs, 710s, 732s, 745s, 775w, 852w,

871m, 912w, 938m, 996m, 1026m, 1070m, 1100vs, 1165s, 1249m, 1283s, 1335m, 1385s, 1433vs, 1450s, 1478s, 1571 m, 1588m, 1682w, 2683w, 2751w, 2849m, 3054s, 3141m. FTIR (ν_{ATR}/cm^{-1}): 306w, 434w, 451m, 473m, 499vs, 507s, 541vs, 588m. Raman (cm^{-1}): 309w, 473w, 639w, 777w, 915w, 1000vs, 1030m, 1072m, 1102m, 1183m, 1289w, 1338w, 1469s, 1585s, 3056vs, 3146w, 3212w, 3345w.

6: ESI+ MS (methanol, *m*/*z*): 721.4 (calc. 721.2; 100%; [Au(PPh$_3$)$_2$]$^+$), 689.1 (calc. 689.0; 5%; {[Au(3*Cl*5*Br*aza)(PPh$_3$)] + H}$^+$), 459.3 (calc. 459.1; 10%; [Au(PPh$_3$)]$^+$), 231.1 (calc. 230.9; 2%; {(H3*Cl*5*Br*aza) + H}$^+$). ESI− MS (methanol, *m*/*z*): 657.3 (calc. 656.8; 100%; [Au(3*Cl*5*Br*aza)$_2$]$^-$), 229.2 (calc. 228.9; 45%; (3*Cl*5*Br*aza)$^-$). FTIR (ν_{ATR}/cm^{-1}): 433m, 456m, 499vs, 541vs, 600m, 615m, 689vs, 710s, 728m, 746s, 781m, 839m, 874m, 927m, 996m, 1010s, 1026w, 1069m, 1099vs, 1147s, 1183s, 1207w, 1249s, 1290s, 1310w, 1330m, 1392vs, 1432vs, 1451s, 1476s, 1532w, 1554w, 1581w, 2682w, 2818w, 2861w, 3051m, 3084w, 3110m, 3143w, 3356w. FTIR (ν_{ATR}/cm^{-1}): 311w, 431m, 447m, 500vs, 542vs, 589m. Raman (cm^{-1}): 278w, 308w, 342w, 447w, 503w, 546w, 618w, 694w, 712w, 731w, 783w, 851w, 931w, 1000vs, 1029m, 1073w, 1103m, 1159w, 1184w, 1262w, 1295w, 1310w, 1333w, 1395w, 1437w, 1456w, 1475m, 1533w, 1586m, 2987w, 3056vs, 3114w, 3142w, 3168m.

7: ESI+ MS (methanol, *m*/*z*): 780.9 (calc. 780.9; 3%; {[Au(3*I*5*Br*aza)(PPh$_3$)] + H}$^+$), 721.4 (calc. 721.2; 100%; [Au(PPh$_3$)$_2$]$^+$), 459.2 (calc. 459.1; 10%; [Au(PPh$_3$)]$^+$), 323.1 (calc. 322.9; 2%; {(H3*I*5*Br*aza) + H}$^+$). ESI− MS (methanol, *m*/*z*): 840.9 (calc. 840.7; 100%; [Au(3*I*5*Br*aza)$_2$]$^-$), 321.2 (calc. 320.8; 30%; (3*I*5*Br*aza)$^-$). FTIR (ν_{ATR}/cm^{-1}): 444w, 501vs, 523m, 543s, 595m, 690vs, 709s, 747s, 774w, 842m, 876m, 929m, 985s, 1026w, 1071w, 1100vs, 1154m, 1181s, 1250m, 1288s, 1329m, 1381vs, 1433vs, 1453s, 1479m, 1577, 2889w, 3013w, 3050m, 3072w, 3100w. FTIR (ν_{ATR}/cm^{-1}): 319w, 343w, 436w, 446w, 500vs, 523s, 543vs, 589w, 596m. Raman (cm^{-1}): 279w, 285w, 321w, 346w, 435w, 500w, 600w, 618w, 693w, 712w, 778m, 931w, 1000vs, 1029m, 1071w, 1102m, 1160w, 1183w, 1252w, 1301m, 1332w, 1384w, 1436w, 1458m, 1532w, 1586s, 3019w, 3055vs.

8: ESI+ MS (methanol, *m*/*z*): 721.4 (calc. 721.2; 100%; [Au(PPh$_3$)$_2$]$^+$), 625.2 (calc. 625.1; 10%; {[Au(2*Me*4*Cl*aza)(PPh$_3$)] + H}$^+$), 459.3 (calc. 459.1; 5%; [Au(PPh$_3$)]$^+$), 167.1 (calc. 167.0; 2%; {(H2*Me*4*Cl*aza) + H}$^+$). ESI− MS (methanol, *m*/*z*): 527.6 (calc. 527.0; 100%; [Au(2*Me*4*Cl*aza)$_2$]$^-$), 165.2 (calc. 165.0; 20%; (2*Me*4*Cl*aza)$^-$). FTIR (ν_{ATR}/cm^{-1}): 441w, 501s, 542s, 616w, 647w, 690vs, 713m, 744vs, 781w, 804m, 859m, 959m, 996m, 1024m, 1072w, 1103vs, 1150m, 1180m, 1247s, 1294vs, 1324s, 1372m, 1398vs, 1434vs, 1480m, 1516m, 1539m, 1583s, 1664w, 2690w, 2845w, 2898m, 2935w, 2975m, 3011m, 3056m, 3086w, 3161w, 3278m, 3386s. Raman (cm^{-1}): 269w, 340w, 361w, 403w, 538m, 618w, 647w, 694w, 713w, 758w, 859w, 924w, 960w, 999vs, 1028m, 1071w, 1103m, 1160w, 1183w, 1260w, 1302w, 1330w, 1372w, 1399w, 1439w, 1479w, 1525vs, 1586s, 2849w, 2902w, 2925w, 2955w, 2988w, 3011w, 3052vs, 3109w, 3141w, 3168w.

Table S1. The parameters of selected non-covalent contacts in the crystal structure of the complexes [Au(*3I5Br*aza)(PPh_3)] (**7**) and [Au(*2CH*$_3$*4Cl*aza)(PPh_3)]·½H_2O (**8′**).

D–H···A	d(D–H)	d(H···A)	d(D···A)	<(DHA)	Symmetry Codes
Complex **7**					
C21–H21A···I1 ^{i}	0.95	3.11	3.818(4)	132.3	(i) −x, 1 − y, −z
C15–H15A···Br1 ^{ii}	0.95	2.95	3.783(4)	147.3	(ii) 1 − x, 0.5 + y, 0.5 − z
C31–H31A···Br1 ^{iii}	0.95	2.99	3.738(4)	137.1	(iii) 1 − x, 1 − y, 1 − z
C25–H25A···C11 ^{iv}	0.95	2.77	3.613(5)	148.4	(iv) 1 + x, y, z
C33–H33A···N7 ^{v}	0.95	2.68	3.362(5)	129.1	(v) 2 − x, 1 − y, 1 − z
C34–H34A···N7 ^{iv}	0.95	2.71	3.538(5)	146.2	(iv) 1 + x, y, z
C35–H35A···Cg ^{iv}	0.95	2.61	3.396(4)	140.4	(iv) 1 + x, y, z
C33···C35 ^{v}			3.326(6)		(v) 2 − x, 1 − y, 1 − z
Complex **8′**					
O1–H1W···N7	0.81(3)	2.17(3)	2.976(3)	170(3)	
C15–H15A···C3A ^{vi}	0.95	2.79	3.602(4)	143.8	(vi) 1.5 − x, 1.5 − y, 1 − z
C21–H21A···C33 ^{vii}	0.95	2.73	3.635(4)	158.5	(vii) x, 1 − y, z − 0.5
C34–H34A···C22 ^{viii}	0.95	2.90	3.518(4)	124.1	(viii) 1 − x, y, 0.5 − z
C35–H35A···O1	0.95	2.56	3.496(3)	166.6	

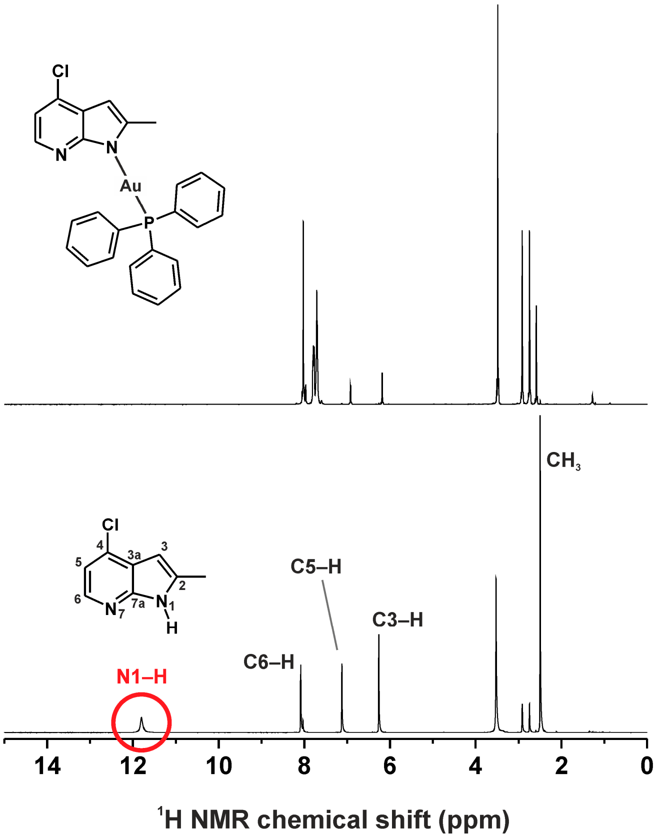

Figure S1. ^{1}H-NMR spectra (DMF-*d*$_7$) of H*2Me4Cl*aza (**bottom**) and [Au(*2Me4Cl*aza)(PPh_3)] (**8**; **top**) proving the deprotonation of the used 7-azaindole derivative; DMF = dimethylformamide.

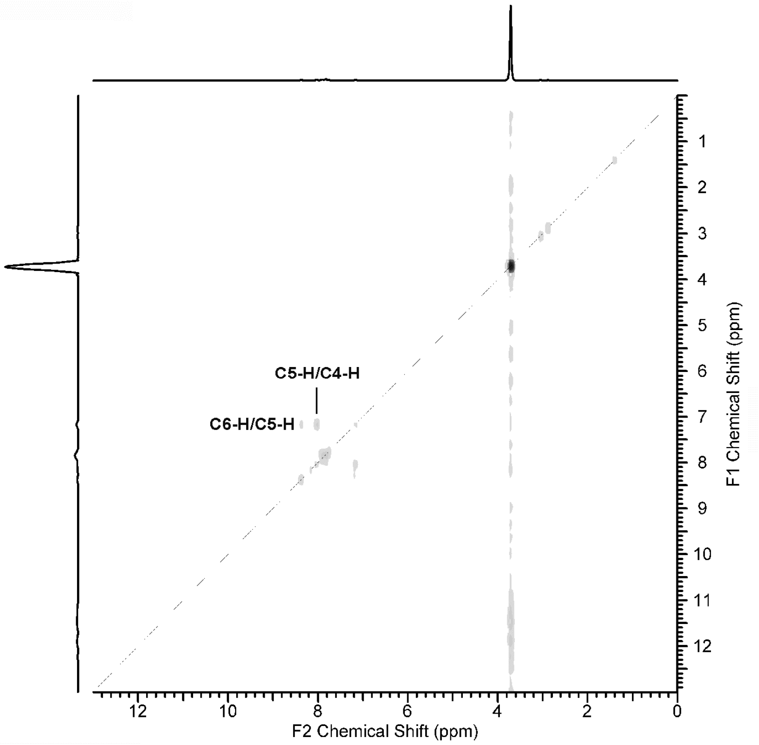

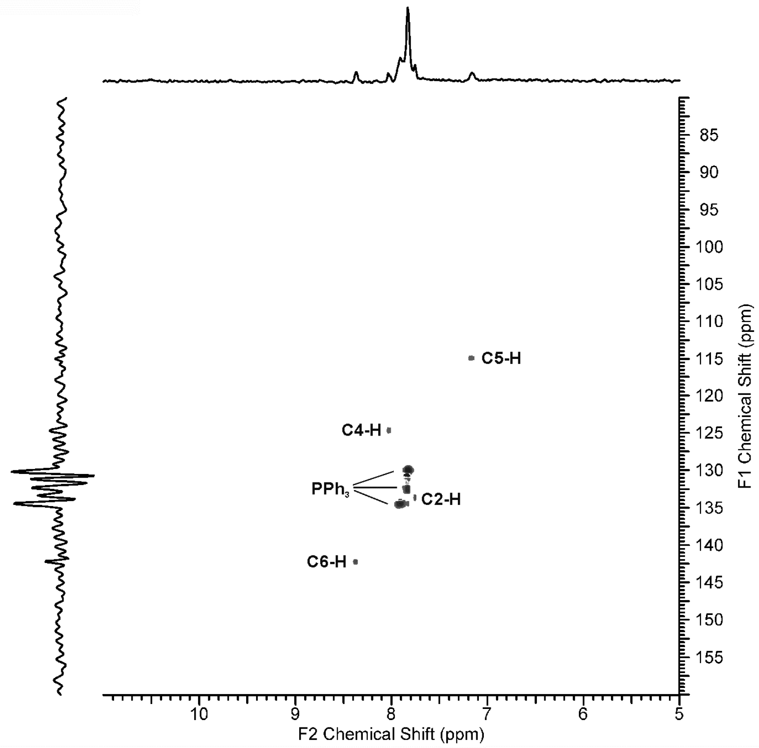

Figure S2. ^{1}H–^{1}H gsCOSY (top) and ^{1}H–^{13}C gsHMQC (**bottom**) spectra of the representative complex [Au(*3ClBr*aza)(PPh_3)] (**2**).

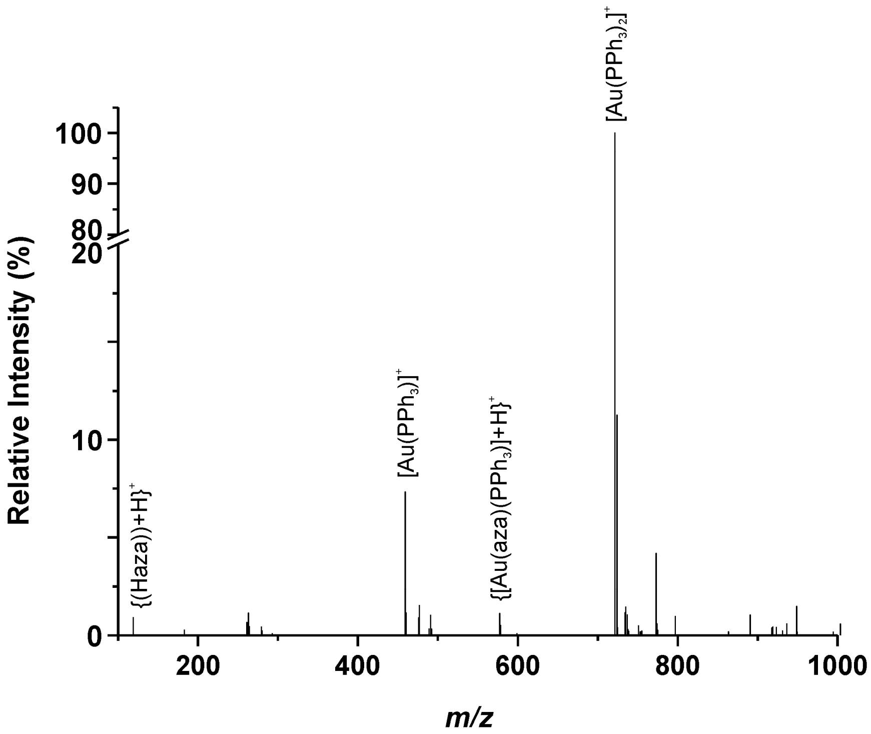

Figure S3. ESI+ mass spectrum of the representative complex [Au(aza)(PPh3)] (**1**).

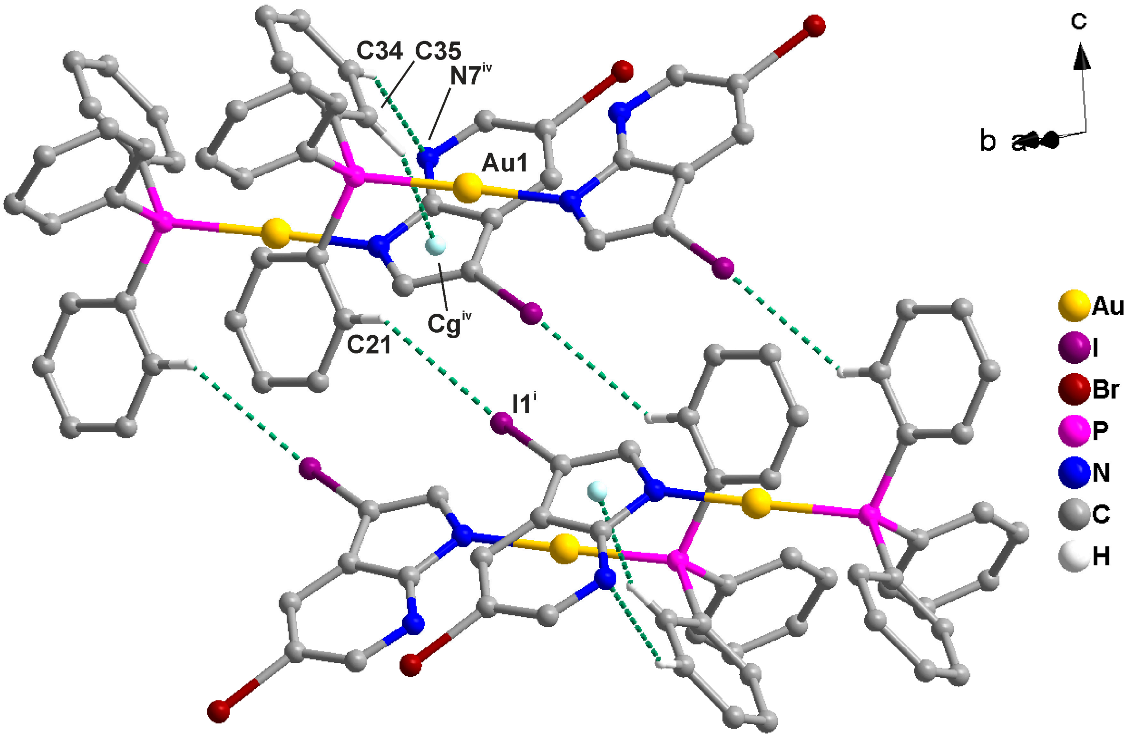

Figure S4. Part of the crystal structure of [Au(*3I5Br*aza)(PPh3)] (**7**) with depicted selected C–H···I, C–H···N and C–H···π non-covalent interactions (see Table S1 for parameters) with the hydrogen atoms not involved in the system of non covalent contacts omitted for clarity. Symmetry codes: (i) −x, 1 − y, −z; (iv) 1 + x, y, z.

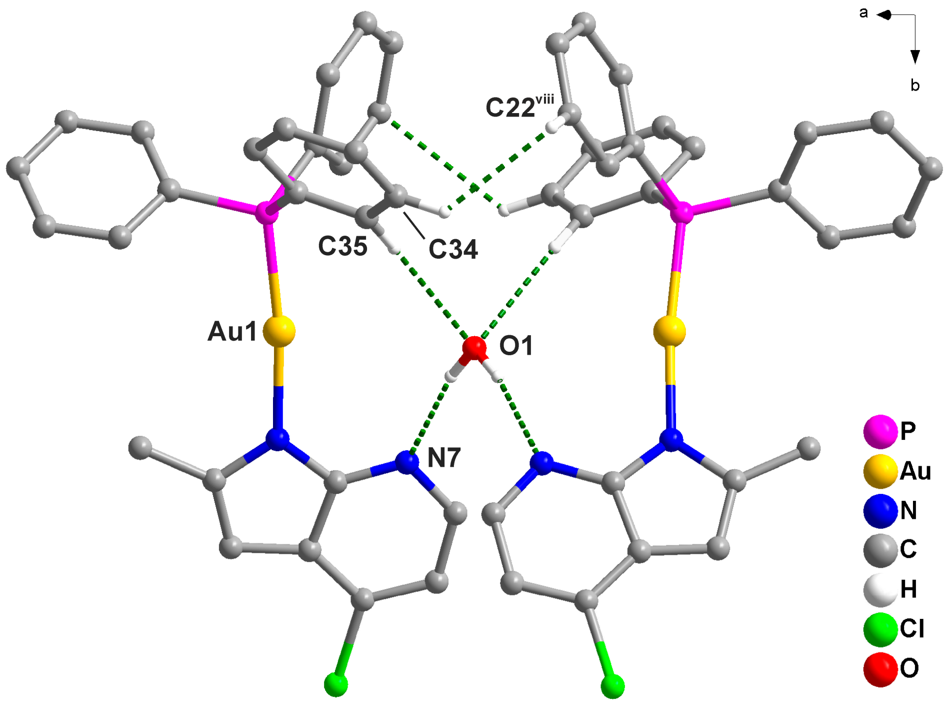

Figure S5. Part of the crystal structure of [Au(2*CH*3*4Cl*aza)(PPh3)]·½H2O (**8′**) with depicted selected O–H···N, C–H···O and C–H···C non-covalent interactions (see Table S1 for parameters) with the hydrogen atoms not involved in the system of non covalent contacts omitted for clarity. Symmetry codes: viii) 1 − x, y, 0.5 − z.

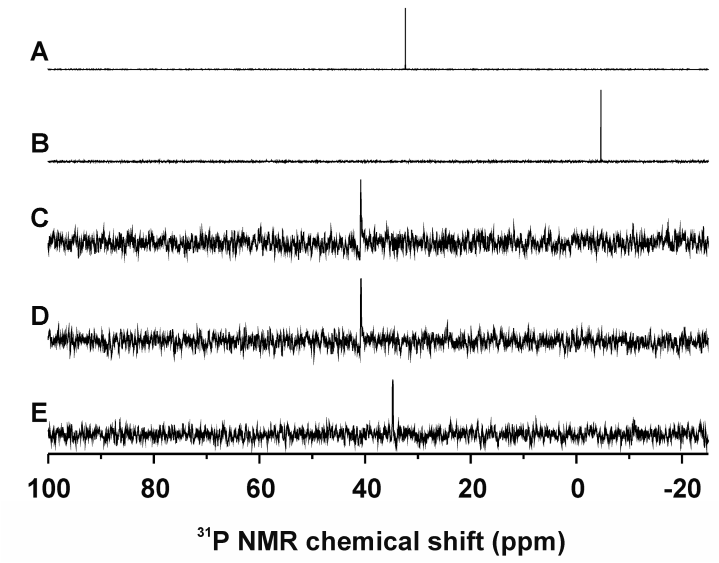

Figure S6. The results of ^{31}P-NMR spectroscopy performed for oxidized triphenylphosphane (O = PPh$_3$; **A**), triphenylphosphane (PPh$_3$; **B**), the mixture of complex **8** and 1 (**C**) or 5 (**D**) mol equiv of reduced glutathione (GSH), and complex **8** (**E**). The samples were dissolved in the 50% DMF-*d*$_7$/50% D$_2$O mixture.

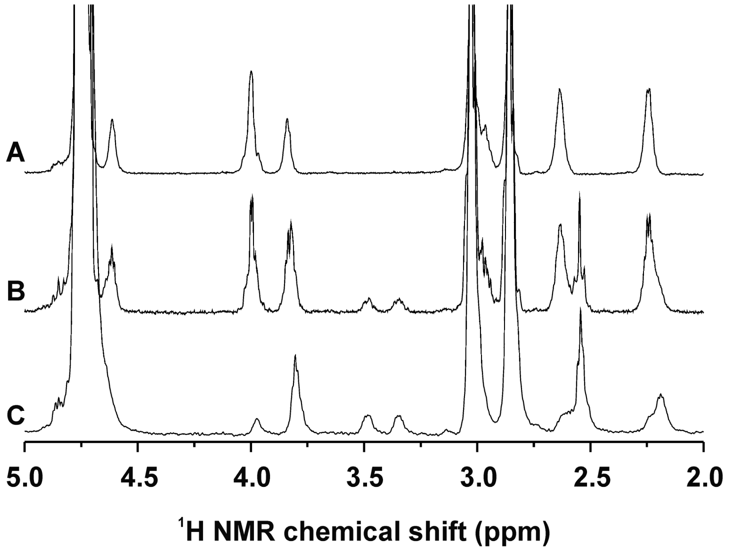

Figure S7. (**A**) ^{1}H-NMR spectrum of reduced glutathione (GSH); (**B**) the ^{1}H-NMR spectroscopy results of the interaction experiment of complex **8** and 5 mol equiv of GSH; (**C**) the ^{1}H-NMR spectroscopy results of the interaction experiment of complex **8** and 1 mol equiv of GSH. The samples were dissolved in the 50% DMF-*d*7/50% D_2O mixture.

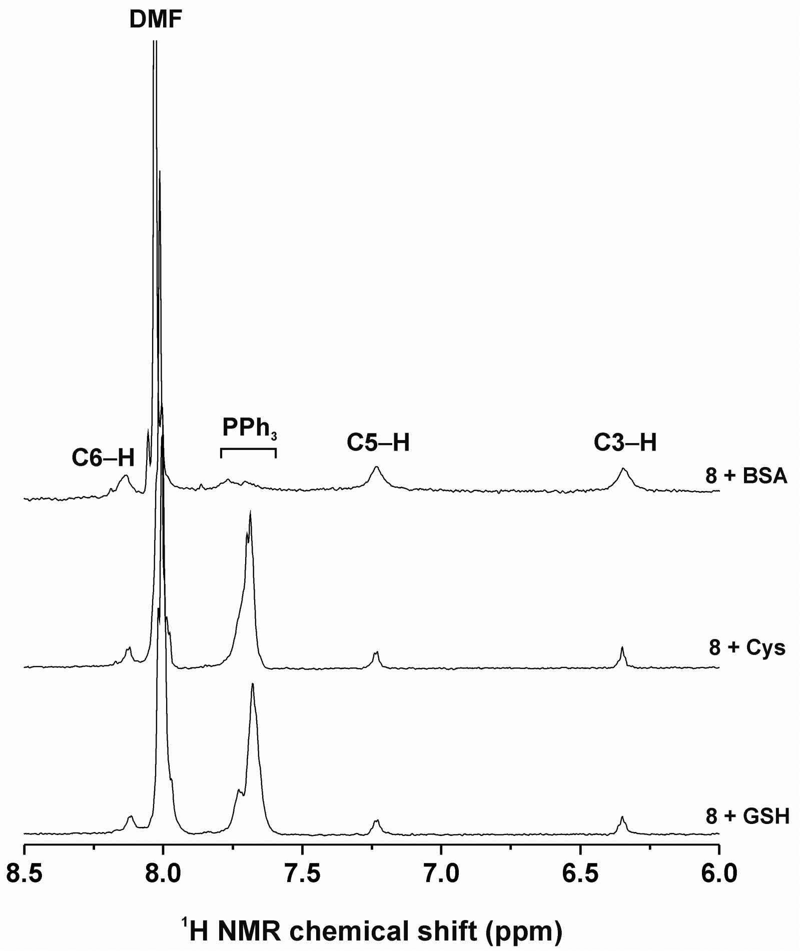

Figure S8. ^{1}H-NMR spectra of the equimolar mixtures of the representative complex **8** with reduced glutathione (**8** + GSH), cysteine (**8** + Cys) and bovine serum albumin (**8** + BSA). The samples were dissolved in the 50% DMF-*d*7/50% D_2O mixture.
